# Delayed Contrast Extravasation MRI for Depicting Tumor and Non-Tumoral Tissues in Primary and Metastatic Brain Tumors

**DOI:** 10.1371/journal.pone.0052008

**Published:** 2012-12-14

**Authors:** Leor Zach, David Guez, David Last, Dianne Daniels, Yuval Grober, Ouzi Nissim, Chen Hoffmann, Dvora Nass, Alisa Talianski, Roberto Spiegelmann, Zvi R. Cohen, Yael Mardor

**Affiliations:** 1 Oncology Institute, Sheba Medical Center, Ramat-Gan, Israel; 2 Advanced Technology Center, Sheba Medical Center, Ramat-Gan, Israel; 3 Neurosurgery Department, Sheba Medical Center, Ramat-Gan, Israel; 4 Radiology Institute, Sheba Medical Center, Ramat-Gan, Israel; 5 Pathology Institute, Sheba Medical Center, Ramat-Gan, Israel; 6 Sackler Faculty of Medicine, Tel-Aviv University, Tel-Aviv, Israel; Beijing Tiantan Hospital, Capital Medical University, China

## Abstract

The current standard of care for newly diagnosed glioblastoma multiforme (GBM) is resection followed by radiotherapy with concomitant and adjuvant temozolomide. Recent studies suggest that nearly half of the patients with early radiological deterioration post treatment do not suffer from tumor recurrence but from pseudoprogression. Similarly, a significant number of patients with brain metastases suffer from radiation necrosis following radiation treatments. Conventional MRI is currently unable to differentiate tumor progression from treatment-induced effects. The ability to clearly differentiate tumor from non-tumoral tissues is crucial for appropriate patient management. Ten patients with primary brain tumors and 10 patients with brain metastases were scanned by delayed contrast extravasation MRI prior to surgery. Enhancement subtraction maps calculated from high resolution MR images acquired up to 75 min after contrast administration were used for obtaining stereotactic biopsies. Histological assessment was then compared with the pre-surgical calculated maps. In addition, the application of our maps for prediction of progression was studied in a small cohort of 13 newly diagnosed GBM patients undergoing standard chemoradiation and followed up to 19.7 months post therapy. The maps showed two primary enhancement populations: the slow population where contrast clearance from the tissue was slower than contrast accumulation and the fast population where clearance was faster than accumulation. Comparison with histology confirmed the fast population to consist of morphologically active tumor and the slow population to consist of non-tumoral tissues. Our maps demonstrated significant correlation with perfusion-weighted MR data acquired simultaneously, although contradicting examples were shown. Preliminary results suggest that early changes in the fast volumes may serve as a predictor for time to progression. These preliminary results suggest that our high resolution MRI-based delayed enhancement subtraction maps may be applied for clear depiction of tumor and non-tumoral tissues in patients with primary brain tumors and patients with brain metastases.

## Introduction

MRI is considered the gold standard imaging modality for the central nervous system. Although MR images provide high resolution and good sensitivity standard MR sequences still suffer of low specificity especially in the case of brain tumors during or after treatment. Tumor progression, inflammation and infection can induce similar changes in enhancement which are impossible to differentiate. Treatment related changes, including radiotherapy-induced changes, add another level of complexity to the interpretation of the brain images which remains challenging notwithstanding the increased utilization of advanced imaging methodologies. Previous studies suggest that nearly half of glioblastoma multiforme (GBM) patients with radiological deterioration after standard chemoradiation do not suffer from tumor recurrence but from pseudoprogression induced by the treatment [1]–[8]. Treatment decision, as whether to operate on a patient with radiographic deterioration, continue chemoradiation or change to another non-surgical treatment is a daily struggle involving interdisciplinary teams of neurosurgeons, neuro-oncologists and neuro-radiologists which are often unable to reach unanimous interpretation of the patient’s status. Therefore, reliable distinction between these conditions is essential for appropriate patient management and for appropriate patient selection for clinical trials [9]–[13].

It has been previously suggested [14]–[16] that the histological tumor fraction (i.e., tumor burden) comprises a subcomponent of the total enhancement seen on contrast-enhanced MRI and represents a potentially useful predictor of survival in patients with recurrent brain tumors. In fact, studies suggest that histological quantification of tumor burden provides more meaningful prognostic information than simply reporting the presence of tumor [17]–[18].

Brain metastases are the most common intracranial tumor in adults, occurring in approximately 10% to 30% of adult cancer patients. It is believed that the annual incidence is rising (due to better treatment of systemic disease and improved imaging modalities). The prognosis of patients diagnosed with brain metastases is generally poor.

**Table 1:** Biopsied samples and map characteristics.

Sample #	Patient #	delayed enhancement population	Histological description	Tumor type
1	1	Mixed regions of red, blue and green populations	One cellular region consisted of small cells with no mitoses. Proliferation was seen in 5% of the cells by Ki67 staining, implying active tumor. Other regions showed post radiation changes. One region was of brain parenchyma with no obvious abnormalities	GBM post chemoradiation
2	1	Cortical region of blue population and deeper white matter region of red population	Subcortical infiltrating zone of active tumor with rare mitosis and a deeper, white matter region of post radiation changes. Ki67 staining of the active tumor zone showed proliferation in 3–5% of the cells	GBM post chemoradiation
3	1	Region of blue population	Active tumor consisting of a hypercellular area of small cells. Ki67 staining showed proliferation in 10–12% of the cells	GBM post chemoradiation
4	1	Mixed regions of blue and red populations	Regions of active tumor consisting of a hypercellular area of small cells and regions of post radiation changes. Ki67 staining in the active tumor region showed proliferation in 10–12% of the cells	GBM post chemoradiation
1	4	Region of red population	Radiation necrosis	GBM post chemoradiation
2	4	Cortical region of blue population and white matter region of red population	Cortical region shows active tumor accumulating focally beneath the meninges. Focal proliferation of blood vassals and palisading necrosis are identified as well. Most of the deeper white matter region show radiation necrosis	GBM post chemoradiation
1	11	Region of blue population	Highly cellular tumor with small regions of tumor necrosis with and without pseudo palisading regions of proliferating blood cells	Secondary GBM post chemoradiation
2	11	Region of blue population	Highly cellular tumor	Secondary GBM post chemoradiation
3	11	Region of blue population	Highly cellular tumor with small regions of tumor necrosis with and without pseudo palisading regions of proliferating blood cells	Secondary GBM post chemoradiation
4	11	Region of blue population	Highly cellular tumor with large proliferating vessels and small regions of tumor necrosis with palisading regions of proliferating blood cells	Secondary GBM post chemoradiation
1	13	Region of blue population	Tumor consisting of atypical cells, mitoses and proliferating vessels	Newly diagnosed GBM
1	14	Region of blue population	Tumor consisting of atypical cells and numerous mitoses	GBM post chemoradiation
2	14	Border between a red regionand a blue region	A region of necrosis with scanty nuclear dust and a region of tumor with pleomorphism and small regions of palisading necrosis	GBM post chemoradiation
1	16	Border between a red regionand a blue region	Necrotic region including necrotic blood vessels and nuclear dust and a cellular tumor region with atypical cells, mitoses and vascular proliferation	Newly diagnosed GBM
2	16	Region of blue population	Tumor region with small foci of palisading necrosis	Newly diagnosed GBM
1	17	Region of blue population	Highly cellular tumor with small necrotic foci	Newly diagnosed GBM
1	19	Region of blue population	Regions of high cellularity and of low cellularigy typical of oligodendroglioma tumors	Newly diagnosed analplastic oligodendroglioma
1	20	Region of red population on theborder of a blue population	Mostly necrotic tissue including necrotic blood vessels. Small foci of tumor are present	Newly diagnosed analplastic oligodendroglioma
2	20	Region of blue population on theborder of a red population	Most of the tissue is tumor. One small area of necrosis at the periphery of the section	Newly diagnosed analplastic oligodendroglioma
3	20	Region of blue population on theborder of surrounding brain	Mostly tumor tissue bordered by brain tissue infiltrated by tumor	Newly diagnosed analplastic oligodendroglioma
1	21Metastasis #1	Sample taken from a blue region bordered by a green region on one side and a red region on the other	A sample showing a region of morphological active tumor bordered by normal cerebellum tissue on one side and necrotic tissue on the other	Sample taken from NSCLC cerebellar brain metastasis
2	21Metastasis #1	Sample taken from a red region bordered by a blue region	A sample showing a large necrotic region borders by tumor	Sample taken from NSCLC cerebellar brain metastasis
1	27	Mixed area of blue and red regions	A mixture of active tumor regions and necrotic regions	Sample taken from NSCLC cortical brain metastasis
2	27	Blue region with small red foci on the border of surrounding brain	Several small samples of active tumor, tumor necrosis and edematous brain	Sample taken from NSCLC cortical brain metastasis
1	29	Red region on the border of surrounding brain	Radiation induced gliotic brain tissue	Sample taken from breast cerebellar brain metastasis
2	29	Mixed blue and red region on the border of surrounding brain	cerebral tissue and mixed regions of tumor and radiation necrosis	Sample taken from breast cerebral brain metastasis
3	29	Mixed blue, green and red regions	tumor, cerebral tissue and radiation necrosis with ecstatic blood vessels	Sample taken from breast cerebral brain metastasis
4	29	A red region bordered by surrounding brain on one side and a blue region on the other	Gliotic brain and radiation necrosis with a small tumor mass	Sample taken from breast cerebral brain metastasis
1	30	Red region surrounded by a blue rim	small regions of tumor (∼30% of the sample) within larger region of necorosis (∼70% of the sample)	Sample taken from breast cortical brain metastasis
2	30	Blue region on the border of surrounding brain	small tumoral region on the border of normal cortex	Sample taken from breast cortical brain metastasis
3	30	Blue region on the border of surrounding brain	Highly cellular tumor adjacent to normal cortex	Sample taken from breast cortical brain metastasis
4	30	Red region bordered by small blue region on one side and surrounding brain on the other	mostly necrosis with small foci of tumor and adjacent normal brain	Sample taken from breast cortical brain metastasis

List of 32 biopsied samples, delayed enhancement subtraction map characteristics and histological evaluation of 9 patients with primary brain tumors and 4 patients with brain metastases.

Stereotactic radiosurgery (SRS) is a radiotherapy technique which permits the delivery of a single large dose of radiation to the tumor while minimizing irradiation of adjacent normal tissue. It is applied to treat both benign and malignant tumors as well as for vascular lesions and functional disorders. Among the reported complications of SRS is radiation-induced necrosis which, similarly to pseudoprogression, can be difficult to differentiate both clinically and radiologically from recurrent tumor at the treatment site. The incidence of radiation induced necrosis may vary between 5% to 11% according to the volume of the treated lesion and the applied dose [19].

The difficulty in interpretation of these post treatment images prevents appropriate treatment in nearly half of treated GBM patients and 5–11% of treated brain metastases patients [1]–[8], [19]. The ability to clearly differentiate tumor from non-tumoral tissues is crucial for appropriate patient management in GBM and brain metastases as well as in other brain tumors undergoing surgery, chemotherapy and/or radiation therapy.

Conventional MRI is currently unable to provide reliable distinction between tumor progression and treatment effects such as pseudoprogression and radiation necrosis [9]–[11]. MRS can distinguish residual or recurrent tumors from pure treatment-related necrosis, but not from mixed necrosis and tumor tissue [20]. Diffusion weighted MRI (DWMRI) and diffusion-tensor MRI has also been assessed for differentiating tumor/necrosis after RT [21]–[26], however, the specificity of DWMRI is less than MRS. It has been suggested that combining DWMRI with MRS may improve the differentiation [27]. FDG-PET has been shown to be useful in differentiating necrosis from recurrence, but the reported sensitivity and specificity of FDG PET in the brain are low [28]. There is limited, but increasing evidence that PET with amino acid tracers may contribute to the differentiation between treatment-related necrosis from tumor recurrence [29]. Whether these techniques will also allow a reliable distinction between pseudoprogression and real progression is yet to be determined.

**Figure 1:**
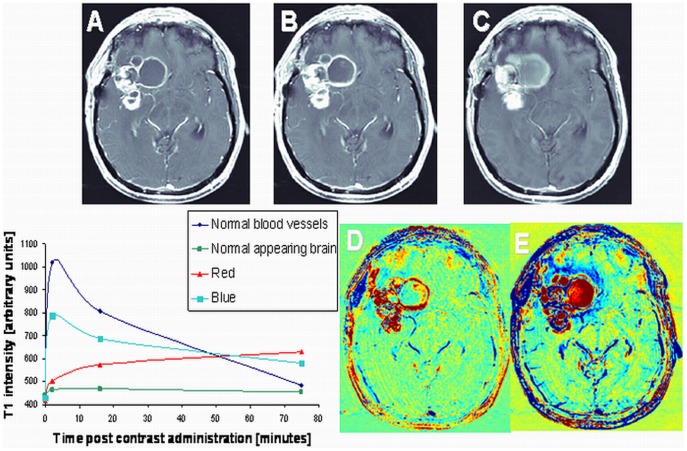
Enhancement subtraction maps. Examples of axial high resolution T1-weighted MR images acquired 2 min (A), 15 min (B) and 75 min (C) after contrast administration in a patient (#3) with newly diagnosed GBM undergoing standard chemoradiation are shown. Subtraction maps were calculated from the data acquired at 2 and 15 min (D) and 2 and 75 min (E) post contrast administration. Blue regions represent fast clearance of the contrast agent from the tumor while red regions represent slow accumulation of the contrast in the tissue. It can be seen that abnormal enhancement patterns in the 75 min map are depicted more clearly and over larger regions than in the 15 min map. The signal intensity of regions with different enhancement patterns as a function of time post contrast administration is shown in the plot. It can be seen that the red and blue components of the tumor enhance and decay at different rates.

**Figure 2:**
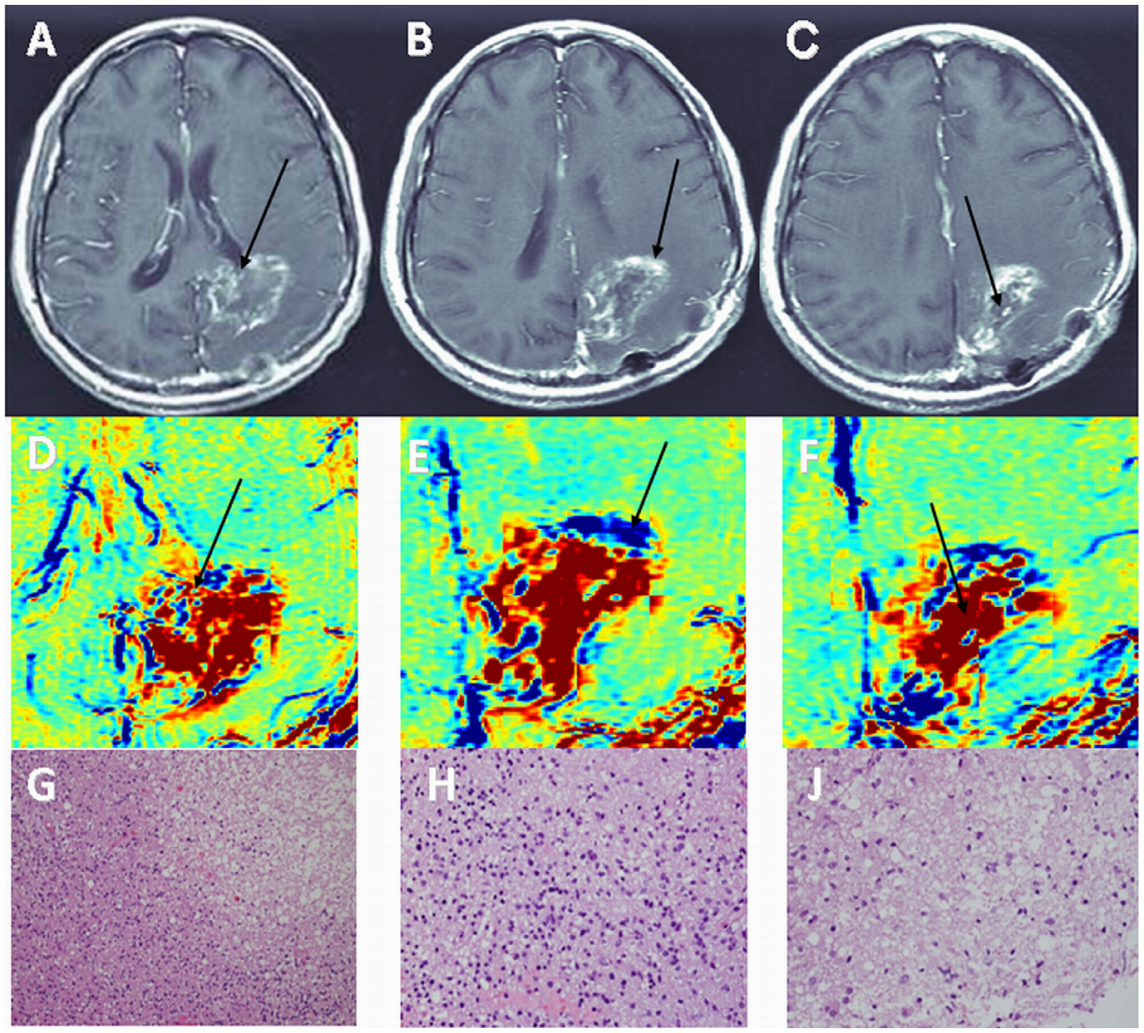
Histological determination of tumor and non-tumoral components – GBM. Examples of contrast-enhanced T1-weighted MRI (A–C), enhancement subtraction maps calculated from the 2 and 75 min data (D–F) and H&E stained histological samples of a rapidly growing lesion in patient #1 with newly diagnosed GBM undergoing standard chemoradiation are shown. Data was acquired prior to surgery, 6 months after initiation of treatment. Samples were taken from a mixed blue and red region (A, D, arrows), a blue region (B, E, arrows) and a red region (C, F, arrows). Histological analysis reveals mixed regions of tumor and necrosis (G, magnification×200), hypercellular tumor (H, magnification×400) and radiation necrosis (J, magnification×400), respectively.

**Figure 3:**
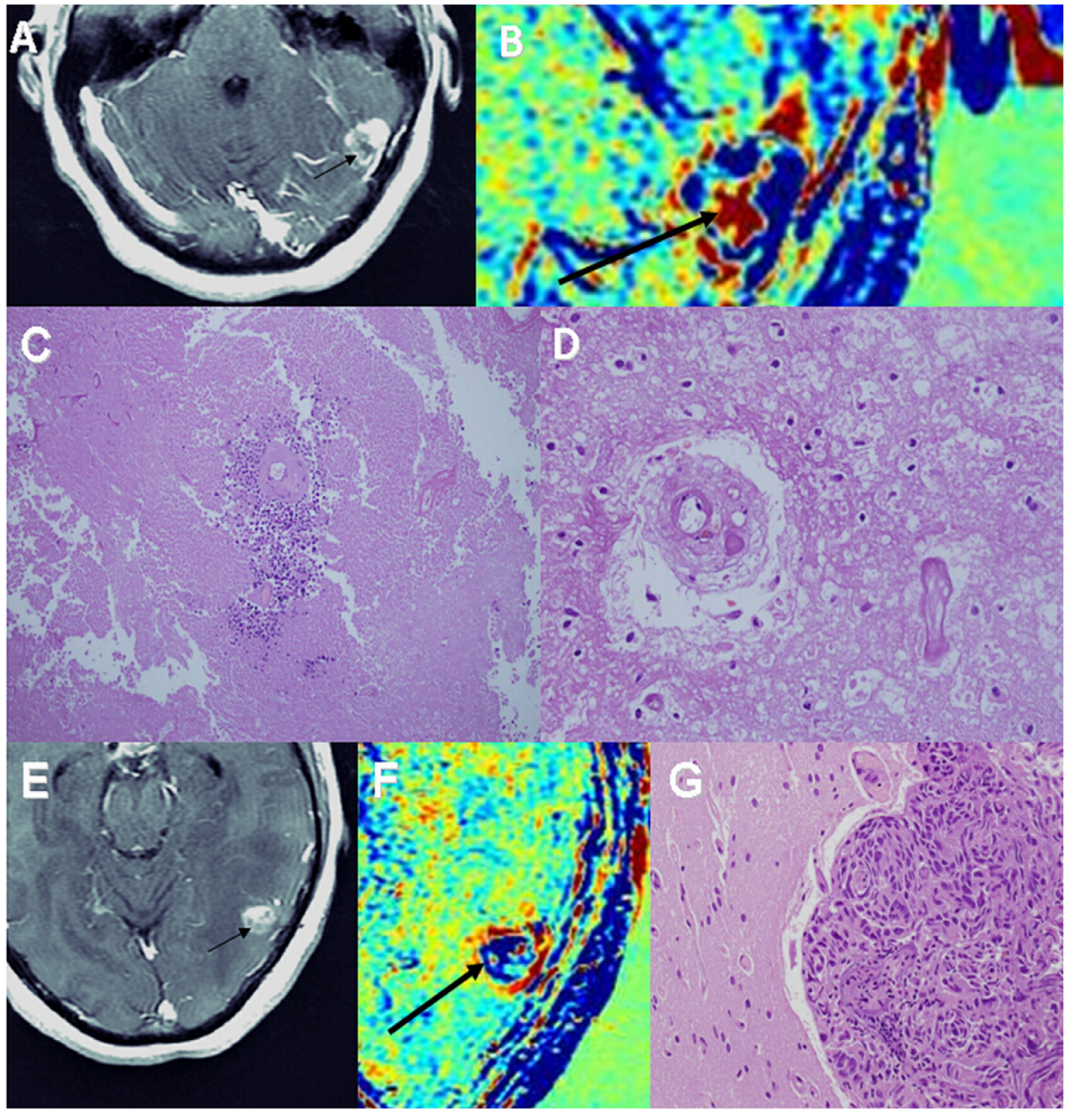
Histological determination of tumor and non-tumoral components – brain metastases. Examples of contrast-enhanced T1-weighted MRI (A, E), enhancement subtraction maps calculated from the 2 and 75 min data (B, F) and H&E stained histological samples (C, D, G) of a cortical breast cancer brain metastasis of patient #30, 2 years post radiosurgery, are shown. Sample #4 taken from a red region marked by arrows in A and B shows a small tumor foci, surrounding a viable blood vessel, within a larger region of necrosis (magnification x100). An example of necrotic blood vessels within the necrotic region (x400) is shown in D. Sample #3 taken from a blue region on the border of normal brain marked by arrows in E and F shows a highly cellular tumor adjacent to normal cortex (G, x200).

A number of fast acquisition MRI techniques have been applied to study microvasculature parameters in this context. The two most commonly used methods are dynamic contrast-enhanced MRI (DCE MRI) and dynamic susceptibility-weighted contrast MRI (DSC MRI) [30]–[32]. DCE MRI measures the changes in T1 relaxation associated with disrupted BBB following contrast administration using parameters such as fractional blood volume (fBV) and permeability (Kps or Ktrans). DSC MRI uses echo planar sequences with a rapid bolus of gadolinium-based contrast agents to assess changes in T2* within the vasculature and interstitial space. Typical calculated parameters are the relative peak height (rPH), relative cerebral blood volume (rCBV) and the percentage recovery (%REC) or recirculation factor (RF).

**Figure 4:**
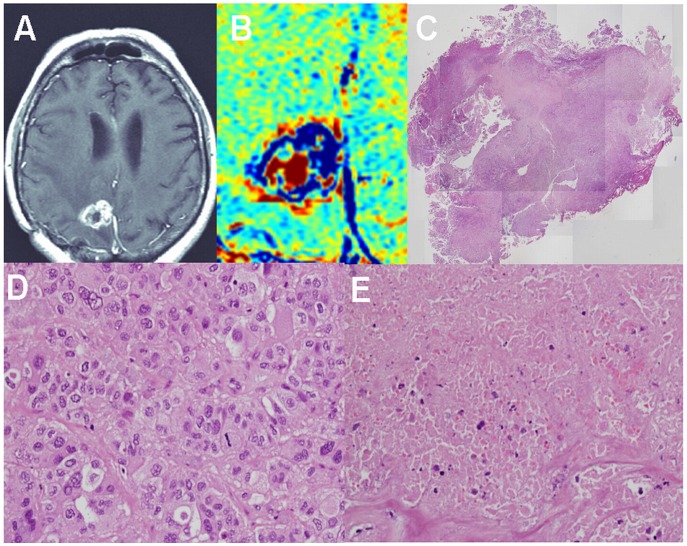
Histological determination of tumor and non-tumoral components – brain metastases. Examples of contrast-enhanced T1-weighted MRI (A), enhancement subtraction map calculated from the 2 and 75 min data (B), macro H&E stained histological sample (C, magnification x20), tumor region from a peripheral region of the sample (D, magnification x400) and radiation necrosis from the central region of the sample (E, magnification x400) of a medial NSCLC brain metastasis of patient #23 (metastasis #1), 1 year post radiosurgery, are shown. The metastasis was resected unblock and marked by the neurosurgeon to enable comparison with the MRI data. H&E staining shows a large central necrotic region surrounded by a rim of morphologically active tumoral tissue, in agreement with the subtraction map. It is also possible to see part of a necrotic blood vessel in the region of radiation necrosis (E) and scattered blood cells in the tissue.

**Table 2:** Non-biopsied tumors histology and map characteristics.

Patient #	delayed enhancement population	Histological description*	Tumor type
3	Overall enhancing lesion consists of71% blue population and22% red population	Cellular tumor with many mitoses and regions of “geographic necrosis”. In some regions of the tumor it is also possible to see proliterative blood vesselsSome regions surrounding the tumor (not in all slices and not all around the tumor) depict brain tissue infiltrated by a small number of tumor cells. The main findings in these regions around the tumor are abnormal proliferation of blood vessels and many histiocytes	GBM post chemoradiation
21 Metastasis #2	lesion consisting of blue (58%)regions and red (31%) regions	Metastatic carcinoma showing squamoid features with extensive areas of tumor necrosis	NSCLC cortical brain metastasis
22	lesion consisting of blue (56%)regions and red (35%) regions	Several samples showing regions of tumor and regions of radiation necrosis. Significant cauterize artifacts are noticed as well	Adenoid Cystic Carcinoma Cortical brain metastasis
23 Metastasis #1	Metastasis consisting of a centralred (42%) region surrounded by athick blue rim (47%)	Central slice shows a large necrotic region surrounded by significant regions of morphologically active tumor	NSCLC mediall brain metastasis Pathology report addressing metastases resected unblock
23 Metastasis #2	lesion consisting of blue (56%)regions and red (33%) regions	Morphologically active tumor is present in the histological samples	NSCLC cortical brain metastasis
24	lesion consisting of a large blue (56%)region surrounded by a red rim (34%)	Active tumor was found	Melanoma midline brain metastasis
25	lesion consisting of a blue central region(61%) surrounded by a red (33%) region	Active tumor was found	Breast cortical brain metastasis
26	lesion consisting of thin blue rim(40%) within a larger red (51%) mass	Large mass of radiation necrosis. Small fociof active tumor were found after ki67 staining	Breast peri-ventricular brain metastasis
28	lesion is mostly red (53%) with smallelongated blue regions (27%)	Mostly necrotic samples with small foci of active tumor	NSCLC medial brain metastasis

List of tumors with no stereotactic biopsies, delayed enhancement subtraction map characteristics and histological evaluation of 1 patient with primary brain tumor and 7 patients with brain metastases.

* Pathology report is based on all samples obtain from the neurosurgeons unrelated to the pre-surgical maps.

Parametric maps that are derived from DCE and DSC data have been proposed as noninvasive methods for assessing response to therapy. Treatment induced changes typically shows decreased rCBV, whereas recurrence shows high rCBV [33]–[39]. Still, most of these studies show some degree of overlap between the two disease entities. DSC was recently applied by Gahramanov et al [40] which demonstrated the feasibility for differentiating pseudoprogression from real tumor progression using ferumoxytol. Narang et al [41] applied DCE to a cohort of 29 patients with gliomas and brain metastasis suspected for treatment-induced necrosis or recurrent/progressive tumor and demonstrated the feasibility of predicting real progression. Barajas et al [42] applied DSC MRI for differentiating tumor progression from radiation necrosis in GBM patients undergoing external beam radiation therapy. Their analysis showed that rPH and rCBV were significantly higher in patients with recurrent GBM than in patients with radiation necrosis while the %REC values were significantly lower.

Using fast acquisition techniques has the disadvantages of low spatial resolution and high sensitivity to susceptibility artifacts. Due to these limitations, significant results are mostly obtained by averaging over the entire enhancing lesion while important information regarding the location and shape of small active tumor regions may be limited. This effect stands out especially in the case of GBM, where psuedoprogression hardly ever describes a status with no residual tumor (unlike the case of brain metastases and radiation necrosis where complete tumor resolution is more likely to occur), as we know that the vast majority of all GBM patients, including those experiencing pseudoprogression, eventually recur.

More encouraging results were obtained using delayed T1-weighted MRI (T1-MRI) permeability methods, which image beyond the first pass circulation of contrast, sometimes as long as 10–15 min. Hazle et al were able to reliably distinguish between recurrence, radiation necrosis, and a combination of both factors [43]. They found that radiation necrosis and tumor enhance at different rates, enabling significant differentiation between recurrent tumor, radiation necrosis and mixed radiation necrosis and tumor (p<0.001). Diehn et al [44] showed that using intra-tumoral and peri-tumoral MRI information it was possible to predict activation of hypoxia and proliferation gene-expression programs, respectively. Furthermore, the intratumoral distribution of gene-expression patterns was found to predict patient outcome.

**Figure 5:**
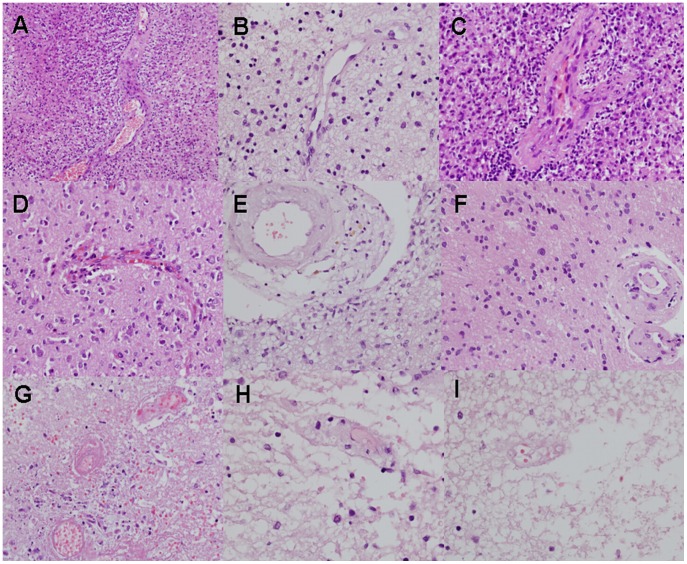
Vessel morphology. Examples of vessel morphology sampled from regions appearing blue in the maps of patients with primary brain tumors are shown in images A–F. Vessels from regions appearing red in the maps are shown in G–I. Samples D and G were taken from patient #4. Samples B, E, H and I were taken from patient #1. A and C were taken from patient #11 and F was taken from patient #13. It can be seen that the samples obtained from blue regions in the maps (A–F) present swollen endothelial cells, dilated lumen, peri-vascular dense fibrous tissue and glomeruloid lumen. Samples taken from red regions in the maps show different stages of vessel necrosis. The vessels shown in G show early necrosis, with scattered blood cells surrounding the necrotic vessels, while the vessels in H and I show later stages of vessel necrosis. The silhouette is reserved and there are residual red blood cells but the endothelial cells are necrotic.

**Figure 6:**
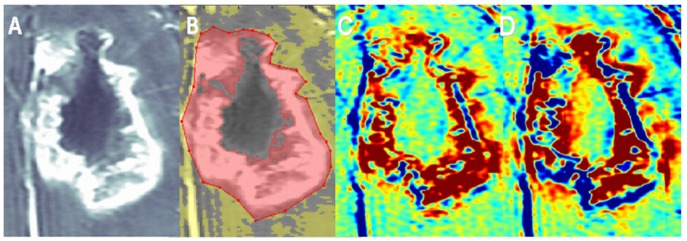
Enhancing lesion volume. Contrast-enhanced T1-weighted MRI without (A) and with (B) a mask selecting the enhancing portion of a GBM tumor (patient #4) are shown. The enhancing lesion volume was calculated from the pixels marked pink in (B). Enhancement subtraction maps calculated at 15 min (C) and 75 min (D) demonstrate the contributions of the red/non-tumor and blue/tumor contributions to the enhancing lesion volume.

In the current study we apply a novel methodology based on delayed contrast extravasation MRI for calculating delayed enhancement subtraction maps, depicting unique temporal enhancement characteristics with high resolution and high sensitivity to subtle BBB disruption [45]. In order to confirm the application of our maps for differentiating tumor tissues from non tumoral tissues we compared pre-surgical maps of patients with primary or metastatic brain tumors with histological assessment of resected tissue samples.

## Materials and Methods

### Patients and Treatment

The study was conducted after approval of the local ethics committee at Sheba Medical Center. Written informed consent was obtained from all patients.

Ten patients with primary brain tumors and 10 patients with brain metastases were recruited prior to surgery and scanned by conventional and delayed contrast extravasation MRI. In all 20 patients pre-surgical delayed enhancement subtraction maps were compared with histological findings. In addition, the application of our maps for prediction of progression was studied in a small cohort of 13 newly diagnosed GBM patients undergoing standard chemoradiation and followed up to 19.7 months post therapy.

### Primary Brain Tumors Group

The primary brain tumor patients consisted of 8 patients with histologically confirmed glioblastoma (World Health Organization [WHO] grade IV astrocytoma) of which 3 were newly diagnosed patients, 4 progressed following chemoradiation (60 Gy in 30 daily fractions, 5 days a week, with concomitant daily temozolomide of 75 mg/m^2^ for 42 days. Chemoradiation was followed by adjuvant temozolomide of 150–200 mg/m^2^ daily for 5 days every 28 days.) and one patient with secondary GBM was recruited 2 years after resection of an Anaplastic Astrocytoma (WHO grade III tumor) and chemoradiation. Two patients with newly diagnosed Anaplastic Oligodendrioma (WHO grade III tumor) were recruited as well.

**Figure 7:**
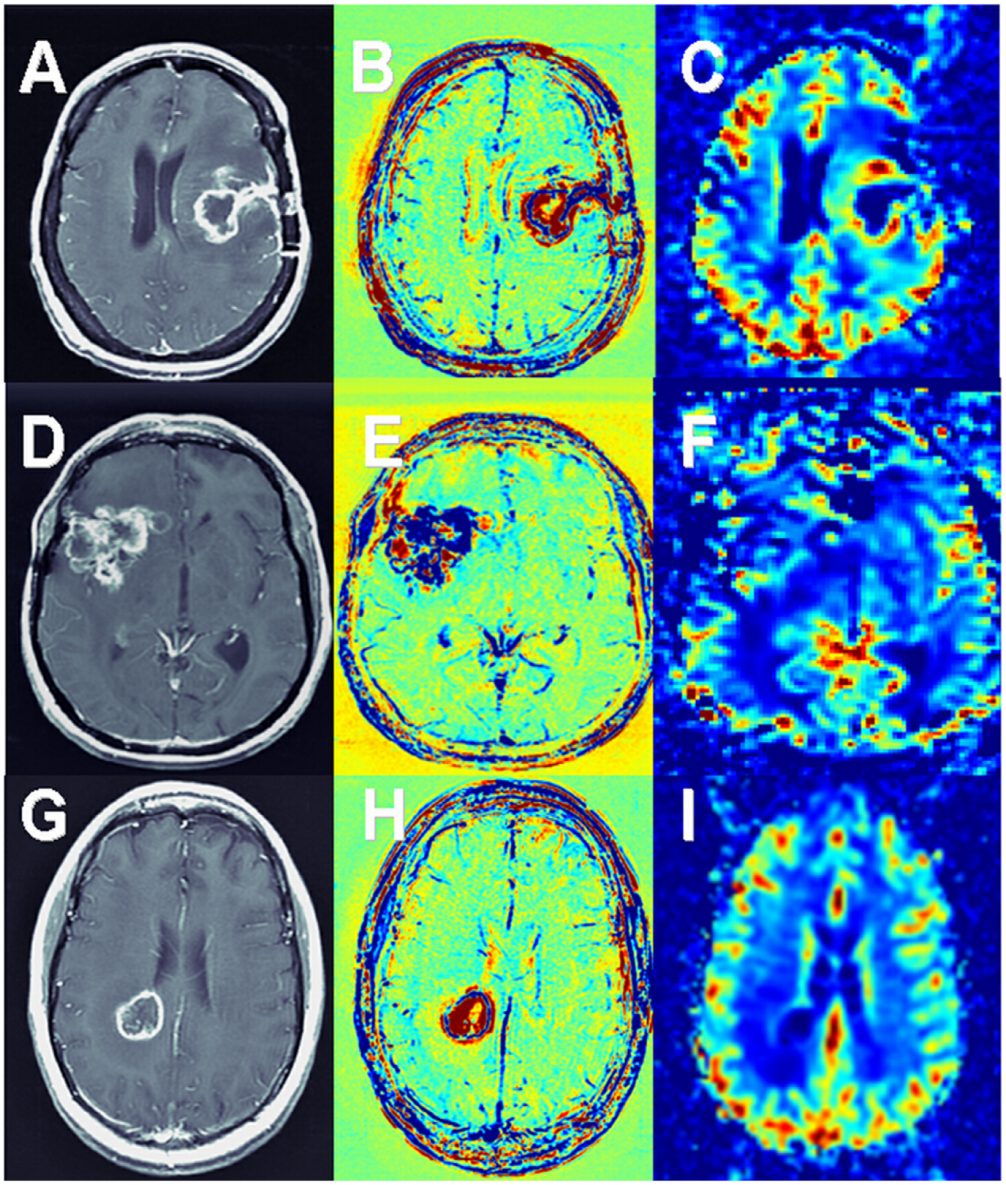
Comparison with rCBV. Contrast-enhanced T1-weighted MRI (A, D, G), enhancement subtraction maps (B, E, H) and rCBV maps (C, F, I) of patients # 6 (A–C), #3 (D–F) and #26 (G–I) are shown. Patient #6 (GBM) shows a blue rim surrounding the surgery site, representing morphologically active tumor, in agreement with high rCBV values in the corresponding rCBV map. Patient #3 (GBM) is a contradicting example, showing a massive lesion dominated by the blue population in the subtraction maps (confirmed by histology to consist of ∼70% morphologically active tumor), in contrast to low rCBV values in the corresponding rCBV map. Patient #26 (breast cancer brain metastases) shows a thin rim of the blue populations in our maps in agreement with a thin rim of increased rCBV values. The advantages of our vessel function maps over rCBV acquired using DSC in means of high resolution, high sensitivity to contrast and minimum sensitivity to susceptibility artifacts can be seen.

**Figure 8:**
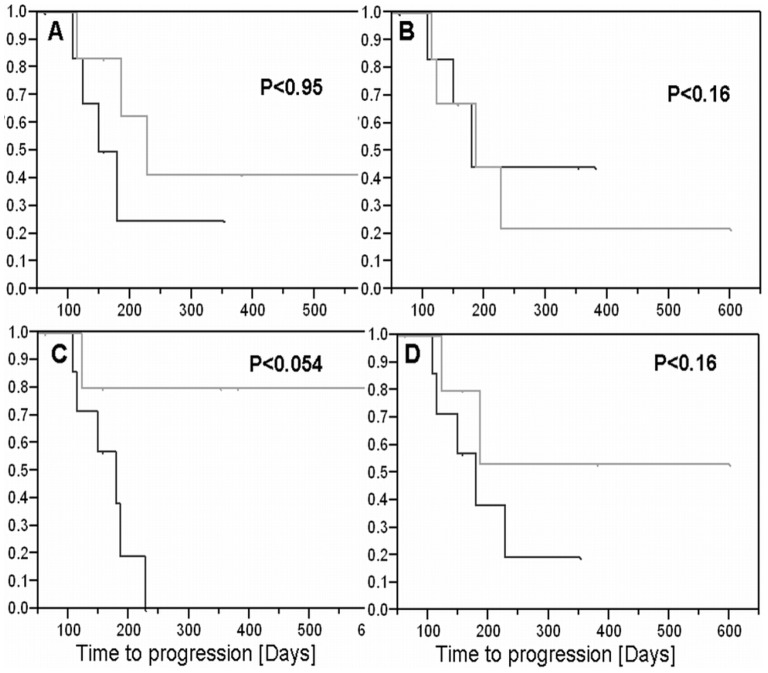
Correlation with time to progression. The correlation between the late enhancement subtraction maps and time to progression was studied in a small cohort of 13 GBM patients post chemoradiation. Kaplan-Meier curves of time to progression in patients above and below the median of four predictors are shown: Initial fast volume (A), initial enhanced volume (B), initial fast growth rate (C) and initial enhanced growth rate (D). The curves are plotted for each predictor for patients above (black) and below (gray) the median. It can be seen that the initial fast growth rate predictor provides a near-significant difference between the two groups of patients, suggesting this predictor may be a candidate for prediction of time to progression.

The mean age of this group of patients was 58.6±3.6 with a range of 37–80. Nine of the 10 patients were men.

### Brain Metastases Group

The brain metastases patients consisted of 4 patients with breast cancer metastases, 4 with non small cell lung cancer (NSCLC) metastases, 1 with malignant melanoma metastases and 1 patient with adenoid cystic carcinoma metastases. All patients were treated with a single dose of 18–20 Gy (to the 80% isodose line) LINAC based SRS 13.1±2.9 months prior to resection.

The mean age of this group of patients was 50.2±4.0 with a range of 30–67. Four of the 10 patients were men.

**Table 3:** Newly diagnosed GBM cohort.

Patient #	1^ST^ Surgery	2^nd^ Surgery	Samples comparedwith histology	Time to progression [months]	Treatment at progression
1	GTR	STR	4	7.5	Surgery+Bevacizumab
2	GTR	–	–	Not reached (19.7)	–
3	GTR	GTR	8	6.5	surgery
4	GTR	GTR	2	6.2	Surgery+Bevacizumab
5	STR	–	–	3.6	Bevacizumab
6	GTR	GTR	–	11.6	Surgery+ Bevacizumab
7	GTR		–	Not reached) 12.5	–
8	STB		–	Died from unrelated disease (3)	–
9	STB		–	Not reached (5.2)	–
10	GTR		–	5.0	Bevacizumab
12	STB		–	4.1	Bevacizumab
15	GTR		–	3.6	Bevacizumab
18	GTR		–	Not reached (5.2)	–

List of newly diagnosed GBM patients with post chemoradiation treatment follow-up.

* Column #2: Patients were diagnosed with GBM prior to initiation of chemoradiation by histological analysis of either gross tumor resection (GTR), sub-total resection (STR) or stereotactic biopsy samples (STB).

* Column #4: Stereotactic samples were taken from locations determined using the late enhancement subtraction maps calculated from the pre-surgical MRIs.

* Column #5: In cases where progression was not reached, the duration of follow-up is listed in parenthesis.

**Figure 9:**
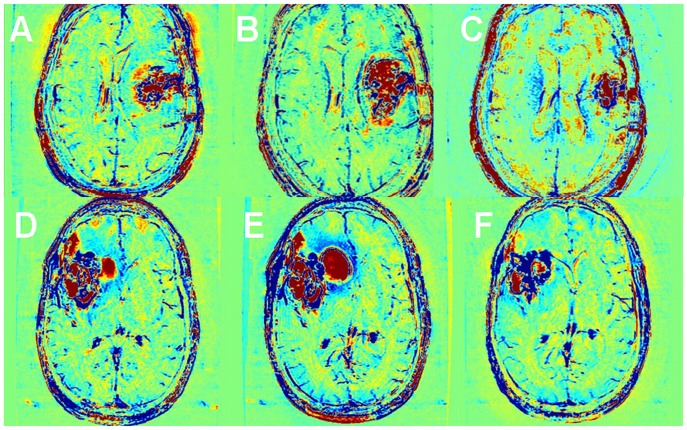
Examples of progression and pseudoprogression in GBM patients post chemoradiation. Late enhancement subtraction maps of a patient (#6) with significant increase in the enhancing lesion due to increase in the red volume (A–C) and a patient (#3) with significant increase in the blue component (D–F) with minor changes in the enhancing volume are shown. In the first example, the total enhancing volume has increased by 34% from 3 weeks (A) to 4.2 months (B) post chemoradiation, and then decreased to 33% below the initial volume (C) 9 months post treatment. The blue volume slightly increased by 6% in the first 4 months (A, B) and then significantly decreased to 47% below the initial volume at 9 months (C) while the red volume increased by 51% in the first 4.2 months (A, B) and decreased to 13% above the initial volume by 9 months (C). This patient progressed 11.6 months post treatment. In the second example, the total enhancing volume has increased by 16% from 3 weeks (D) to 2.5 months (E) and then remained 17% above the initial volume (F) 6.5 months post treatment. The blue volume slightly increased by 2% in the first 2.5 months (D,E) and then significantly increased to 57% above the initial volume at 6.5 months (F) while the red volume increased by 39% in the first 2.5 months (D, E) and decreased to 61% below the initial volume by 6.5 months (F). This patient progressed 6.5 months post treatment when he was referred to surgery.

### Newly Diagnosed GBM Group

In an attempt to explore additional possible applications of our calculated maps, 13 newly diagnosed GBM patients were scanned by conventional and delayed extravasation MRI 3–4 weeks after chemoradiation and every 2 months thereafter. Patients with clinical deterioration underwent additional MRI exams at the discretion of the physician. These patients were followed for 2–19.7 months. Eight of the later have progressed while 5 remained progression free.

The mean age of this group of patients was 54.3±4.1 with a range of 28–72. Eight of the 13 patients were men.

Inclusion criteria for all patients included WHO performance status of 2 or less and adequate hematologic, renal, and hepatic function. Exclusion criteria included contraindications to MRI and stable condition to undergo an MRI exam.

### MRI Data Acquisition

MR images were acquired using a clinical General Electric 3.0 T MRI machine (GE Medical Systems, Waukesha, WI, USA) with the HD12 operating system, gradients intensity of up to 4.3 Gauss/cm and the standard GE phased array head-coil. MR sequences included T2* perfusion-weighted MRI (PWI), Fast spin-echo T2-weighted MRI, T2 FLAIR and echo-planar diffusion-weighted MRI (DWMRI). High resolution spin-echo T1-weighted MR images (T1-MRIs) were acquired before and at 3 time points after contrast injection: 2.6±0.1 min (immediately after the PWI sequence), 15.4±0.4 min and 75.3±0.7 on average. These times are referred to as the 2 min, 15 min and 75 min time points throughout the text. In order to minimize the burden on the patients, they were scanned up to 30 min after contrast injection, and were then asked to return for a short scan 75 min after contrast injection. T1-MRI was acquired with TE/TR = 22/240 ms, field of view 26×19.5 cm, 5/0.5 mm slice thickness and 512×512 pixels. A standard single dose (0.2 ml/Kg) of Gd-DOTA (Dotarem, 0.5 mmol/mL, Guerbet, 95943 Roissy CdG Cedex, France) was injected intravenously using an automatic injection system 6 seconds after starting the PWI sequence.

### MRI Data Analysis

All image analysis was performed using MatLab (version R2006b, The MathWorks, Inc. Natick, MA, US).

The overall goal of the analysis was to obtain delayed subtraction maps, where the T1-MRIs of the 1^st^ series post contrast were subtracted from the T1-MRIs of later series. These maps depict spatial distribution of contrast accumulation/clearance in the tissue, blood vessels and CSF. For example, in case of normal blood vessels, due to clearance of contrast agent from the blood system, there is no increase in contrast accumulation in the late scans; therefore, the subtraction maps show negative values (blue in the maps). The signal decay of the blood vessels is faster than that of the tissue (where the signal is averaged over the tissue and microvasculature), therefore, blood vessels have lower values than tissue. In case of contrast accumulation, i.e. regions where contrast clearance is slower than contrast accumulation, the maps show positive values (red in the maps).

In order to increase the sensitivity to small changes it was essential to perform image pre-processing consisting of corrections for intensity variations and whole body image registration.

#### Correcting for intensity variations

Signal intensity homogeneity throughout the image and between slices depends on various parameters including: strength and homogeneity of the static magnetic field, oscillating excitation field, gradients, sensitivity of the receiving coil and various parameters of the sampled tissue. An intensity correction was performed on each image separately by calculating an intensity variation map consisting of the large scale intensity variations. The later map was then subtracted from the original image.

#### Rigid body and elastic/local registration

Rigid body registration was performed using least squares approach and 6 parameter (rigid body) spatial transformation with the SPM5 (Statistical parametric mapping) MatLab routine (an academic software kit by “Wellcome Trust Centre for Neuroimaging”). Since head movements affected the magnetic fields thus inducing distortions in the MRIs, it was necessary to add local/elastic registration. The registration was performed by dividing each slice to a grid of 20×20 mm volumes. Each square volume was allowed to move freely in x-y-z till the sum of the absolute values of the intensity difference between the 2 time points reached a minimum. The resulting three 3D translation matrices were smoothed using circular smearing and interpolated to obtain translation values per pixel. These high resolution matrices were then applied to register T1-MRIs of the second time point to the location of the first time point.

#### Subtraction maps

Following the pre-processing, subtraction maps were calculated by simply subtracting the processed images of the series acquired 2 min after the contrast injection from a series acquired later on.

#### Enhancing lesion volume

The enhancing portion of the lesions, depicted on conventional contrast-enhanced MRI, was calculated from the spin-echo contrast-enhanced T1-MRIs acquired 2 min after contrast injection. Regions of interest (ROIs) were defined over the entire enhancing region in each slice. A threshold, determined from intensity distribution histograms of the tumor and surrounding regions, was applied to the ROIs to include only enhancing portions of the tumor. The number of pixels in the enhancing portions of the ROIs were counted and multiplied by the volume of a single pixel. The resulting volume was referred to as the state of the art parameter for assessment of tumor volume [12].

### Histology

A total of 32 stereotactic tissue samples and 8 resected lesions acquired from 10 patients with primary brain tumors and 10 patients with brain metastases were histologically examined and compared with our delayed enhancement subtraction maps. Stereotactic locations for tissue samples were determined prior to surgery using the calculated subtraction maps which were co-registered to the conventional T1-MRIs, for 9 patients with primary brain tumors (20 tissue samples) and 4 patients with metastatic tumors (12 tissue samples) (Table 1). Existence/absence of morphologically active tumor in the histological analysis of 8 metastatic tumors acquired from 7 patients was compared with the pre-surgical maps as well (Table 2). Eight additional samples were obtained from one GBM patient, chosen by the neurosurgeon during surgery as representative samples (patient #3, Table 2).

All samples were marked by the neurosurgeon during resection and then fixed and stained by H&E according to the routine hospital procedure.

Histological interpretation was performed by the hospital neuro-pathologist. Morphologically active tumor was defined as demonstrating one or more of the following characteristics: hyper cellularity, small cells, mitosis, high Ki67, pseudo-palisading necrosis and vascular proliferation.

Non-tumoral abnormal tissue was defined as demonstrating one or more of the following characteristics: radiation changes including large, widely spaced atypical astrocytes, blood vessels hyalinization, fibrinoid material in vessels, proliferating small vessels and non palisading tumor necrosis.

### Progression (GBM Patients Only)

Disease progression was diagnosed using standard MRI sequences. Each case was presented to the hospital tumor board, consisting of a neuro-oncologist, a neurosurgeon and a neuro-radiologist. The physicians were blinded to the delayed enhancement subtraction maps so that progression was diagnosed according to the RANO (revised Mcdonald’s) criteria. When disease progression was determined, the patient’s treatment was changed (surgery or Bevacizumab). The time from the first MRI follow-up to disease progression is listed for all patients in Table 3. In those patients with no disease progression, the time of the last follow-up was listed.

### Time to Progression (TTP) (GBM Patients Only)

TTP was defined as the time from the end of chemoradiation till progression was determined.

### Tumor Growth Rate (GBM Patients Only)

The feasibility of applying the delayed enhancement subtraction maps for prediction of TTP was demonstrated by studying the correlation between initial tumor growth rate and TTP. Initial tumor growth was calculated as (V−Vo)/Vo, where V was the tumor volume at the second follow-up and Vo was the tumor volume calculated from the first follow-up MRI (3 weeks after the end of treatment). Initial tumor growth rate was calculated by dividing the tumor growth by the time that passed between the first and second MRI follow-ups.

### Statistical Methods

Results for averaging over a group of values are presented as average±standard error. Correlations were assessed by performing t tests and calculating two-tailed p-values, unless otherwise stated. Prediction of TTP was assessed using the logrank test [46]. In view of the small number of patients involved, the p-values were calculated based on the permutation distribution [47]. In this method one calculates the logrank statistic for each possible allocation of the patients to the two predictor groups “high” and “low” preserving the number of patients in each group, and then calculates the p-value as the proportion of allocations that yield a logrank statistic equal to or higher than the one observed with the study data.

## Results

### Delayed Enhancement Subtraction Maps

Enhancement subtraction maps were calculated using the data acquired 15 (15 min maps) and 75 min (75 min maps) after contrast injection. Two primary enhancement patterns were found in our maps: One characterized by slower contrast clearance than contrast accumulation at the delayed time point relative to the 2 min time point (positive signal, colored red in our maps) and the other by faster clearance than accumulation (negative signal, colored blue in our maps). Normal brain regions, due to the intensity variation correction, had an average value of zero (green). Examples of 15 and 75 min maps are shown in Figure 1. It can be seen that the fast (blue) region in the 75 min map is depicted more clearly and over larger regions than in the 15 min map. Examples of the signals intensities of the fast and slow regions as a function of time after contrast injection are shown as well, demonstrating the different rates of contrast accumulation and clearance of these regions.

### Histology, Tumor Versus Non Tumoral Tissues

Thirty two stereotactic samples acquired from 9 patients with primary brain tumors and 4 patients with brain metastases undergoing surgery were compared with our pre-surgery calculated maps. The samples were taken from fast (blue) regions according to the maps, slow (red) regions, and regions consisting of mixed fast and slow components. Histological evaluation confirmed for all samples the discrimination between fast regions, determined to consist of morphologically active tumor, and slow regions, consisting of non-tumoral tissues. Regions consisting of both fast and slow components in the maps consisted of tumor and non-tumoral tissues in the histological samples (Figures 2, 3, 4, Table 1).

Patient #3 (Table 2) with a newly diagnosed GBM underwent a second resection 6 months after chemoradiation due to clinical deterioration. The patient died 10 days post-surgery. The maps calculated from his last MRI scan, showed that the fast component reached 71±3% of the enhancing lesion volume. Histological analysis was performed for 8 samples taken from 2 main regions of the lesion. In both regions the tumor load was estimated by the neuro-pathologist to cover ∼70% of the examined samples, in agreement with our calculated maps.

Eight additional metastases acquired from 7 patients, showed a fast component in our maps and were confirmed by histology to consist of morphologically active tumor (Figures 3, 4, Table 2).

### Histology, Blood Vessels

In an attempt to find a morphological explanation for the difference between the vessels function of fast and slow regions in our maps, we examined the morphological appearance of blood vessels in these regions. Typical fast population vessel morphology consisted of proliferating endothelial cells, dilated lumen, peri vascular fibrosis and glomeruloid vessels. The outline of the vessels lumens in these regions seemed to be undamaged. Vessels in the slow regions, on the other hand, presented different stages of vessel necrosis with significantly damaged lumens. In most vessels a silhouette of the vessel wall could still be recognized, but only rarely residual endothelial cells could still be detected. In some cases scattered blood cells were seen in various distances from the necrotic vessel. Examples are shown in Figures 3, 4, 5.

### Significance of Long Delays

The volumes and intensities of the fast population, calculated from the 75 min maps, were found to be significantly different then those calculated from the 15 min maps (data calculated from 30 MRI exams of the 30 recruited patients): r = 0.91, p<0.0001 and r = 0.79, p<0.0001, respectively (Wilcoxon matched-pairs signed-ranks test). The average ratio between the volumes of the fast population calculated from the 75 min maps and the volumes calculated from the15 min maps was 2.0±0.3 and the average ratio between the intensities of the fast population at the two time points was 1.8±0.1, suggesting increased sensitivity to tumor tissues at the longer delays. There was no significant difference in this increased sensitivity between the primary brain tumors and the brain metastases groups.

### Correlation with Conventional MRI

The volume of the fast population was found to correlate significantly with the enhancing lesion volume (representing the conventional tumor volume): r = 0.94, p<0.0001 (based on 30 acquired MRI exams). According to our maps, in this cohort of patients 48.4±1.9% (on average) of the enhancing lesion on conventional MRI **did not** represent morphologically active tumor (Figure 6). For example, according to our maps only 55%, 38%, 39%, 56% and 83% of the enhancing lesions presented in Figures 1, 2, 6, 7A and 7D respectively, consisted of the fast population. There was no significant difference in the average percentage of the fast population between the primary brain tumors and the brain metastases groups.

Significant correlation was also found between fast population volume×intensity and rCBV calculated from PWI (r = 0.69, p<0.005), suggesting that rCBV may be a dominant characteristic of the fast population. Still, the relatively low r value implies that there may be other contributions to the fast component. Examples of agreement and disagreement between the vessel function maps and rCBV are shown in Figure 7.

### Correlation with TTP (GBM Patients) – Preliminary Results

In an attempt to explore the feasibility of applying our maps for predicting time to progression 13 newly diagnosed patients were followed by conventional and delayed-contrast extravasation MRI. The follow-up periods of each patient are listed in Table 3. Four parameters were studied as possible predictors for progression: Initial fast volume, initial enhancing volume, initial fast growth rate and initial enhancing growth rate. Kaplan-Meier curves of TTP in patients above and below the median of each predictor are shown in Figure 8. The p values were calculated with the log rank test using the permutation distribution. It can be seen that the initial fast growth rate provides a near-significant difference between the two groups of patients, in contrast to the other predictors, suggesting that this predictor may be a candidate for prediction of TTP. A larger study consisting of more patients and longer follow-ups is required in order to assess the application of our maps for this purpose.

### Examples of Progression and Pseudoprogression in GBM Patients Post Chemoradiation

Within the cohort of GBM patients with follow-up, for 7 of the 8 progressing patients, progression was determined in the first MRI follow-up in which significant increase in the fast component volume was noticed. For one patient progression was determined 5 weeks after the increase in the fast component. While an increase in the fast component preceded progression in all patients, significant increase of the enhancing lesion volume was not necessarily followed by progression. An example of a patient who experienced significant increase in the enhancing lesion volume 4.2 months post treatment but remained progression free for an additional 7.4 months is shown in Figure 9A, B, C. An example of a patient who experienced significant increase in the fast volume 6.5 months post treatment (with no significant increase in the enhancing lesion volume) is shown in Figure 9D, E, F. This patient was determined to progress 6.5 months post treatment and underwent surgery.

## Discussion

GBM is the most common and most aggressive type of primary brain tumor in humans. The current standard of care for newly diagnosed GBM is surgical resection (when possible) followed by radiotherapy with concomitant and adjuvant temozolomide chemotherapy. The rate of early treatment induced radiological changes which mimic tumor progression – pseudoprogression - increased significantly since the addition of chemotherapy to the treatment regimen. Due to the increasing occurrence of brain metastases and the expending use of radiosurgery to treat them, the rate of treatment-induced radiation necrosis is rising as well. Conventional MR imaging is currently unable to provide reliable distinction between tumor recurrence and treatment effects. Clinically, this question has important consequences; therefore, a reliable distinction between these conditions is crucial.

A significant advantage of using the delayed enhancement and clearance rates instead of the commonly studied early rates (DCE and DSC) is the ability to apply sequences with lower temporal resolution, such as high resolution spin-echo T1-MRI. These sequences nearly completely avoid susceptibility artifacts while providing high signal-to-noise ratios, high resolution and high sensitivity to contrast variations. By measuring the clearance of the contrast agent from the tissue at these long delays it is reasonable to assume that the sensitivity to physiological parameters is increased, providing additional information unattainable when using short acquisition times. This assumption is supported by the two fold increase in sensitivity to tumoral (fast) tissue obtained by increasing the delay from 15 to 75 minutes. In this context it is important to note that the patients are not held in the scanner for these long times. The patients are scanned for 30 min post contrast injection and then asked to return for an additional short scan of the 75 min point.

Recent PWI studies suggest that high rCBV values are associated with tumor recurrence and low values with treatment effects. Still, up to date, there are no published studies supporting consensuses regarding which PWI-based analytic method best estimates histological tumor fraction as a predictor of progression or overall survival in GBM patients post treatment [17]. Our preliminary results show significant correlation of the fast component with high rCBV values, suggesting that rCBV may be a dominant characteristic of this population. Still, in some patients we observed low rCBV values in fast regions of the subtraction maps. This may be explained by distortion of the PW images in this population of post-surgery patients due to close vicinity of the tumor to surgical screws. Distortion may also be induced by hemorrhages which are frequent in these tumor types. In some cases the contradiction between our maps, depicting a blue component representing morphologically active tumor (confirmed histologically), and low rCBV values may be explained by the low resolution of the PW images impeding the sensitivity to small tumoral regions in contrast to the high resolution/sensitivity of our maps. On the other hand, these contradicting cases may imply existence of other contributions to the fast component, such as increased vessel permeability. A study designed to mathematically model the signal time curves for determining the dominant factors contributing to the different enhancement populations is ongoing.

The most pronounced effect depicted in the delayed enhancement subtraction maps is the clear differentiation between fast and slow populations, where the terms fast and slow refer to the clearance rate of the contrast agent between the early time point (2 min) and the delayed time point (75 min). The common feature of vessels morphology in the fast regions was found to be the undamaged vessels lumens, while vessels in the slow regions presented significantly damaged lumens. Therefore, one explanation for the difference between the 2 populations may be that vessels in the fast regions provide efficient contrast clearance from the tissue, while the damaged lumens in the slow regions are unable to clear the accumulating contrast efficiently, resulting in contrast accumulation.

The association between the fast/slow components of the maps and tumor/non-tumoral tissues as determined by histological analysis is currently based on 32 biopsy samples obtained from 9 patients with primary brain tumors and 4 patients with brain metastases. Additional confirmation was obtained from 1 GBM patient and 8 metastases resected from 7 patients. These data suggest that our subtraction maps, calculated from delayed contrast extravasation MRI, enable clear differentiation between tumor and non-tumoral tissues in various types of brain tumors. Additional data is needed to establish the reliability of this conclusion. This study is ongoing.

The manner of which our maps may be applied for differentiating progression from pseudoprogression in GBM patients post chemoradiation was demonstrated in Figure 9. In our maps, progression is reflected by a significant increase in the fast component of the enhancing lesion while pseudoprogression is reflected by an increase in the slow component with no significant increase in the fast component. Therefore, using our maps in routine MRI follow-up may aid the physician in determining progression versus pseudoprogression in patients presenting an increase in the enhancing lesion on T1-MRI. An increase in the fast component volume, suggesting progression, implies that a change in the current therapy should be employed. No significant increase in the fast volume component, suggesting pseudoprogression, implies that the patient is responding to the current therapy and thus continuation is preferred, if possible.

The maps may be applied in a similar manner to patients following SRS. Patients with growing volumes of the slow component would be recommended for follow-up, if possible, while patients with growing volumes of the fast component would be recommended for treatment such as surgery or repeated SRS.

Due to improved treatment protocols and extended survival early and late radiation-induced neuro-toxicity of patients with brain tumors undergoing radiation-based therapies has become a major concern and efforts to minimize unnecessary exposure of surrounding normal brain without compromising treatment efficacy are of increasing interest [48]–[49]. The ability of our maps to depict morphologically active tumor regions with high resolution may thus be applied for optimizing radiation treatment planning by localizing the treatment to the fast/blue regions in our maps. This methodology may be similarly applied in the post surgery scenario to enable differentiation between post surgical changes and tumor remnants thus allowing depicting and treating residual tumor post surgery.

High resolution depiction of tumoral tissues may also be beneficial in the planning of surgical resections, especially in the case of close proximity to functionally eloquent brain regions. In these cases determination of the exact extent of the tumor might be crucial for the decision whether microsurgical tumor removal might be warranted. Our maps may also be applied for guiding stereotactic biopsies for molecular classification of the tumor. In this case our maps may aid in preventing the acquisition of biopsies with a high amount of necrosis or out of the infiltration zone with ‘contamination’ of the specimen by normal brain tissue, both potentially leading to false-negative results [50].

Novel approaches for local drug delivery including injections, infusions, trans-nasal delivery, convection enhanced delivery, local BBB disruption and various types of polymeric implants may also benefit from the application of our maps for planning and monitoring the treatments.

Increased tumor vascularity has been shown to correlate with both shortened survival and higher grade of malignancy in gliomas. Consequently, anti-angiogenic agents such as Bevacizumab, a monoclonal antibody targeting vascular endothelial growth factor, are now commonly employed to treat progressive malignant gliomas. The wide-spread use of these agents has added a layer of complexity to the evaluation and characterization of malignant gliomas as these agents have been shown to rapidly and markedly decrease contrast enhancement on contrast-enhanced T1-MRI [51]–[54]. We have recently initiated a study in which our subtraction maps are applied to recurrent GBM patients treated by Bevacizumab in an attempt to provide improved depiction of the tumor thus enabling better patient follow-up and understanding of Bevacizumab mechanism of action in GBM patients.

As up to 45% of suspected low-grade gliomas turn out to be high grade gliomas, histological diagnosis is mandatory before any therapeutic decision [55]. As even these partially anaplastic lesions may not display contrast enhancement on conventional contrast-enhanced T1-MRI, additional imaging information is warranted. We hope that the high sensitivity of our maps may provide additional valuable information for delineation of the tumor borders as well as for targeting stereotactic biopsies.

Additional studies are required in order to assess the reliability of these applications.

In summary, the difficulty in interpretation of post treatment images prevents appropriate treatment in nearly half of treated GBM patients and a significant number of patients with brain metastases. The presented results demonstrate the feasibility for a straightforward application of our high resolution delayed enhancement subtraction maps in the daily clinical scenario. The ability to clearly differentiate tumor from non-tumoral tissues may provide the physician with a clear understanding of the patient current situation thus enabling improved patient management.
